# What are the characteristics and where is the highest risk of snakebite accidents in the state of São Paulo?

**DOI:** 10.1590/1980-549720250026

**Published:** 2025-06-02

**Authors:** Gisele Dias de Freitas, Alec Brian Lacerda, Thiago Salomão de Azevedo, Anderson de Oliveira, Roberta Maria Fernandes Spinola, Flávio Santos Dourado, Fan Hui Wen, Francisco Chiaravalloti-Neto

**Affiliations:** I Secretaria de Estado da Saúde de São Paulo, Coordination of Disease Control, “Prof. Alexandre Vranjac” Epidemiological Surveillance Center – São Paulo (SP), Brazil.; II Universidade de São Paulo, School of Public Health – São Paulo (SP), Brazil.; III Secretaria de Saúde do Município de Santa Bárbara d’Oeste – Santa Bárbara d’Oeste (SP), Brazil.; IV Ministério da Saúde, Health Surveillance and Environment Secretariat, General Coordination of Zoonoses and Vector-Borne Diseases Surveillance – Brasília (DF), Brazil.; V Instituto Butantan, Bioindustrial Center – São Paulo (SP), Brazil.

**Keywords:** Snake bites, Animals, poisonous, Venomous snakes, Antivenins, Immunization, passive

## Abstract

**Objectives:**

To understand the pattern of snakebite envenomation, the objective was to describe accidents and deaths by snake genus, age group, sex, race/ethnicity, education level, place of residence and occurrence, seasonality, vegetation cover, and to identify spatial clusters with a higher risk of accidents in the state of São Paulo.

**Methods:**

A descriptive study using data from the Notifiable Diseases Information System (SINAN) on snakebite accidents between 2010 and 2022. The Getis-Ord Gi* index was applied to identify high- and low-risk clusters.

**Results:**

Bothropic accidents predominated (61.5%), affecting men (78.7%), whites (64.7%), adults aged 20-59 years (67.1%), and individuals with low education levels (63.4%). Most accidents occurred in urban areas (55.7%), while deaths were more frequent in rural areas (68.1%), with longer treatment delays. Incidence remained stable, increasing during hot and rainy periods. Many cases were not autochthonous, and vegetation type influenced accident patterns: *Bothrops* in dense and humid areas, *Crotalus* in dry and open regions, and *Micrurus* in both. Spatial analysis highlighted areas of higher and lower risk, varying by accident type.

**Conclusions:**

Identifying the most affected population, seasonality, and high-risk areas provides essential support for preventive actions and effective management. The findings can guide efforts towards vulnerable populations, anticipate preventive strategies during high-incidence periods, and optimize resources, such as professional training and antivenom serum distribution in high-risk regions.

## INTRODUCTION

Snakebite incidents constitute a significant public health concern in Brazil and globally. Each year, an estimated 5.4 million cases and 137,000 fatalities occur worldwide^1^, though actual figures may be even higher^2^. In 2022, Brazil reported 29,543 snakebite cases and 94 related deaths^3^. Within the framework of the “One Health” (*Uma Só Saúde*) approach, addressing this issue requires an integrated strategy that considers the interconnections between human, animal, and environmental health to ensure an effective and comprehensive response^4^.

In Brazil, snakebite incidents have been recorded in the Notifiable Diseases Information System (*Sistema de Informação de Agravos de Notificação* – SINAN) since 1995 but gained greater prominence in 2010 when they were added to the Compulsory Notification List. Currently, snakebites rank among the most frequently reported diseases in the country^3^. Furthermore, in 2017, the World Health Organization (WHO) reinstated snakebites to the list of neglected tropical diseases^5^. In Brazil, medically significant snake species belong to the Viperidae family (*Crotalus* — rattlesnakes, *Bothrops* — jararacas, and *Lachesis* — surucucus) and the Elapidae family (*Micrurus* — coral snakes), which are responsible for crotalic, bothropic, lachetic, and elapid envenomations, respectively^6^.

The state of São Paulo exhibits a diverse range of natural landscapes and climatic conditions, including those found in the Atlantic Forest and Cerrado biomes, which support a wide variety of snake species. Among them, venomous species of the genera *Bothrops*, *Crotalus*, and *Micrurus* are particularly notable, as they are the primary causes of snakebites in the region. Although snakebites have traditionally been more frequent in rural areas, their incidence in urban environments has been increasing. This rise is driven by the unregulated expansion of cities and the accumulation of waste, which attracts rodents — the primary prey of snakes. This scenario underscores the need for effective environmental management and increased public awareness to mitigate the risk of human-snake encounters^7^.

Given these factors and the need to understand the pattern of snakebites in São Paulo, this study aimed to analyze incidents involving venomous snakes and associated fatalities by examining the characteristics of the affected population (age group, gender, race/color, and education level), the species involved, seasonality (dry or rainy periods), and the geographical distribution of cases concerning vegetation cover. Identifying high-risk areas can help guide public health interventions, including preventive strategies, resource allocation, and the planning of healthcare services in priority regions.

## METHODS

A descriptive study on snakebite incidents was conducted in the state of São Paulo between 2010 and 2022, using data extracted from SINAN^8^. The data were selected from reports of accidents caused by venomous animals, including bees, spiders, scorpions, caterpillars, snakes, and other animals. Among the reported snakebite incidents, only those caused by venomous species (bothropic, crotalic, elapid, and lachetic envenomations) were included. Lachetic snakebites were excluded from this study, as no species of this genus are found in the wild in São Paulo^9^.

To assess the sociodemographic characteristics of the individuals affected and the locations of the incidents, the following variables were considered: age, gender, race/color, education level, date of the accident, municipality of residence and occurrence, area of occurrence, time between the accident and medical care, and the outcome of the case.

Incidence, mortality, and lethality rates were calculated according to snake species, age range (0–9, 10–19, 20–59, and 60+), and gender. The annual incidence rate was determined by dividing the number of accidents by the 2016 population, dividing the result by 13 (the number of years in the study period), and multiplying by 1 million. The mortality rate was calculated in the same manner, substituting the number of accidents with the number of deaths. Lethality was calculated by dividing the number of deaths by the number of accidents and multiplying by 100. In the historical series, a logarithmic scale was applied due to the variation in the types of accidents, and for seasonality, the data were represented as percentages for the same reason.

The incidence rates of spatial distribution were calculated using local empirical Bayesian rates^10^, based solely on autochthonous accidents, categorized by the type of accident and municipality of occurrence. Autochthonous accidents were defined as those in which both the municipality of residence and the municipality of occurrence belonged to the same Regional Health Department (RHD). For non-autochthonous accidents, the distance between the municipality of residence and the location of each incident was assessed, calculated using the formula for direct and inverse solutions of geodesics on the ellipsoid^11^.

Demographic and vegetation information was obtained from the Brazilian Institute of Geography and Statistics (*Instituto Brasileiro de Geografia e Estatística* – IBGE). The vegetation covers evaluated were: dense and mixed rainforests, semi-deciduous seasonal forests, mixed vegetation and savannah^12^.

The Getis and Ord index (Gi*) was used to locally analyze the spatial association, based on an indicator of risk concentration for snakebites, with the “false discovery rate” (FDR) adjustment applied^13^. Accidents were categorized according to snake genus and municipality of occurrence. The neighborhood matrix weight was calculated using the fuzzy contiguity spatial weights technique, with the fuzzy contiguity spatial weights from the Pysal package employed to spatially include the municipality of Ilhabela in the state of São Paulo^13^. The results were interpreted by identifying clusters of high and low risk for snakebites.

The software used in this study included GeoDa^14^, Python^15^, Qgis^16^, R-4.3.2^17^, SatScan^18^, and Tabwin^19^.

## RESULTS

Between 2010 and 2022, a total of 27,777 snakebite incidents were recorded in the state of São Paulo. Of these, 17,740 (63.9%) were caused by venomous species, with 61.5% attributed to *Bothrops*, 12.1% to *Crotalus*, and 1.2% to Elapids (Table 1). The incidence of *Bothrops* accidents was 28.8 cases per 1 million population-years, with men being 2.9 times more affected. In adults over 20 years of age, the rate was 1.6 times higher than in children. *Crotalus* accidents had an incidence of 5.6 cases per 1 million population-years, with a male predominance that was three times higher, and 2.7 times higher in adults. *Elapid* accidents registered 0.6 cases per 1 million population-years, with a similar male and adult predominance, at 2.9 times higher (Table 1).

**Table 1:** Distribution of snakebite accidents in the state of São Paulo (2010–2022) by type of accident, gender, age range, incidence and mortality rates (per 1,000,000 inhabitants/year), and annual lethality percentage.

Type of Accident	Gender	Characteristic	Age Range (Years)				Total
			0 To 9	10 To 19	20 To 59	60 Or +	
Bothrops	Male	Incidence	16.51	39.39	53.11	53.61	46.57
		Cases	623	1,456	9,026	2,197	13,302
		Mortality	0.03	0.00	0.11	0.59	0.15
		Deaths	1	0	19	24	44
		Lethality	0.01	0.00	0.02	0.08	0.03
	Female	Incidence	8.36	13.35	12.62	11.63	12.02
		Cases	301	475	2,216	629	3621
		Mortality	0.00	0.00	0.02	0.13	0.04
		Deaths	0	0	4	7	11
		Lethality	0.00	0.00	0.01	0.09	0.02
	Total	Incidence	12.53	26.61	32.53	29.73	28.83
		Cases	924	1,931	11,242	2,826	16,923
		Mortality	0.01	0.00	0.07	0.33	0.09
		Deaths	1	0	23	31	55
		Lethality	0.01	0.00	0.02	0.08	0.03
Crotalus	Male	Incidence	1.96	6.65	10.89	11.22	9.21
		Cases	74	246	1,850	460	2,630
		Mortality	0.03	0.03	0.05	0.17	0.06
		Deaths	1	1	8	7	17
		Lethality	0.10	0.03	0.03	0.12	0.05
	Female	Incidence	1.42	1.74	2.50	1.79	2.15
		Cases	51	62	439	97	649
		Mortality	0.00	0.00	0.00	0.02	0.00
		Deaths	0	0	0	1	1
		Lethality	0.000	0.000	0.000	0.079	0.012
	Total	Incidence	1.70	4.24	6.62	5.86	5.59
		Cases	125	308	2,289	557	3,279
		Mortality	0.01	0.01	0.02	0.08	0.03
		Deaths	1	1	8	8	18
		Lethality	0.062	0.025	0.027	0.110	0.042
Elapidae	Male	Incidence	0.53	0.84	1.00	0.41	0.83
		Cases	20	31	170	17	238
		Deaths	0	0	0	0	0
	Female	Incidence	0.25	0.48	0.38	0.04	0.32
		Cases	9	17	67	2	95
		Deaths	0	0	0	0	0
	Total	Incidence	0.39	0.66	0.69	0.20	0.57
		Cases	29	48	237	19	333
		Deaths	0	0	0	0	0
Total	Male	Incidence	19.00	46.88	65.00	65.25	56.61
		Cases	717	1,733	11,046	2,674	16,170
		Mortality	0.05	0.03	0.16	0.76	0.21
		Deaths	2	1	27	31	61
		Lethality	0.021	0.004	0.019	0.089	0.029
	Female	Incidence	10.03	15.57	15.50	13.46	14.49
		Cases	361	554	2,722	728	4,365
		Mortality	0.00	0.00	0.02	0.15	0.04
		Deaths	0	0	4	8	12
		Lethality	0.000	0.000	0.011	0.085	0.021
	Total	Incidence	14.62	31.52	39.84	35.78	34.99
		Cases	1,078	2,287	13,768	3,402	20,535
		Mortality	0.03	0.01	0.09	0.41	0.12
		Deaths	2	1	31	39	73
		Lethality	0.014	0.003	0.017	0.088	0.027

Source: Notifiable Disease Information System (*Sistema de Informação de Agravos de Notificação* – SINAN).

Given the similarity observed in the patterns of results regarding race/color and education, evaluated separately for each snake genus, the analyses were aggregated across the genera. The annual incidence by race/color was as follows: 20.98 cases per 1 million population-years in whites, 8.7 in mixed-race individuals, 2.1 in blacks, and 0.3 in Asians and indigenous people. In terms of education, the majority of patients had completed elementary school II (18.6%), followed by high school (17.8%) and elementary school I (16.5%). Only 3.5% had higher education, and 1.7% were illiterate. The education level was not reported in 40.4% of the cases. Regarding the location of the accidents, 55.7% occurred in urban areas, 37.6% in rural areas, and 2.1% in peri-urban areas, while 4.6% had no reported location. Concerning the time to treatment, 49.2% were attended to within one hour, 27.2% within 1–3 hours, 9.7% within 3–6 hours, 2.9% within 6–12 hours, 2.1% within 12–24 hours, and 2.8% after 24 hours. In 6.1% of cases, the time to treatment was not recorded.

Among the 17,740 snakebite accidents and 73 deaths (55 caused by *Bothrops* and 18 by *Crotalus*), the mortality rate was 0.1 deaths per 1 million population-years, with an overall lethality rate of 0.03%. In *Bothrops* accidents, mortality was 3.7 times higher in aged individuals compared to adults and 20 times higher than in children. In men, mortality was three times higher, being 4.4 times higher in the aged compared to adults and 8.9 times more frequent than in children. In *Crotalus* accidents, the mortality rate was 0.03 deaths per 1 million population-years, 2.7 times higher in the aged and 4.5 times higher than in children/adolescents. Mortality in men was 19 times higher than in women (Table 1). Due to the similarity in the patterns of deaths, the analyses were grouped. Regarding race/color, 72.2% of the deceased were white, 15.3% were brown, 6.9% were black, and 5.6% had no information. Regarding education, 20.8% had completed elementary school II, 18.1% had completed elementary school I, 4.2% were illiterate, 4.2% had completed high school or higher education, and 48.6% had no information. Of the deaths, 68.1% occurred in rural areas, 18.1% in urban areas, 6.9% in peri-urban areas, and 6.9% had no location information. Regarding the time to care, 31.9% were attended to within one hour, 23.6% within 3–6 hours, 15.3% within 1–3 hours, 12.5% after 24 hours, 6.9% within 12–24 hours, 4.2% within 6–12 hours, and 5.6% had no recorded time (Table 1).

**Figure 1:**
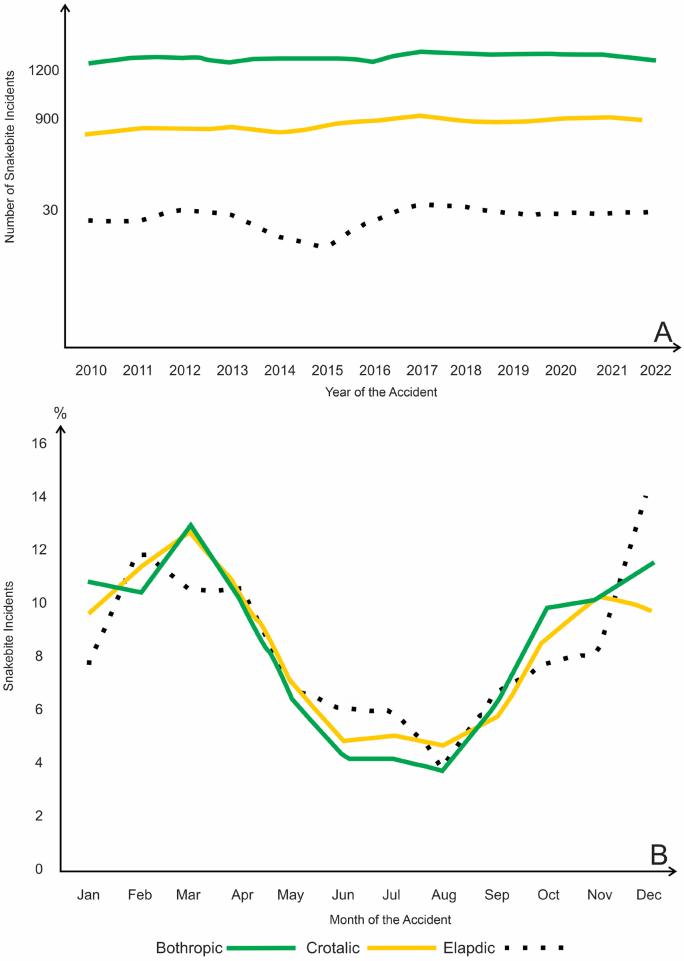
Distribution of the number* of snakebite accidents in the state of São Paulo (2010–2022), according to snake genus, year (temporality), and month (seasonality) of occurrence.

Reports of *Bothrops* and *Crotalus* accidents remained stable over time, while *Elapid* accidents declined in 2015 (Figure 1A). During the São Paulo winter (June to August), fewer reports of *Bothrops* and *Elapid* accidents were recorded, while *Crotalus* accidents remained more consistent (Figure 1B). In *Bothrops* accidents, 2016 saw the highest number of deaths (7), followed by 2019–2021 (6/year) and 2011 (5 deaths). March had the highest number of deaths (15), followed by September–November (6/month). In *Crotalus* accidents, the years 2011, 2012, and 2017 recorded the highest number of cases (3/year), with February showing the highest frequency (four deaths).

*In the historical series (A), a logarithmic scale was used to present the number of accidents due to the large difference in amplitude between the three types of accidents. In the seasonality (B), the representation was in percentages for the same reason.

Source: Notification Disease Information System (*Sistema de Informação de Agravos de Notificação* – SINAN).

**Figure 2:**
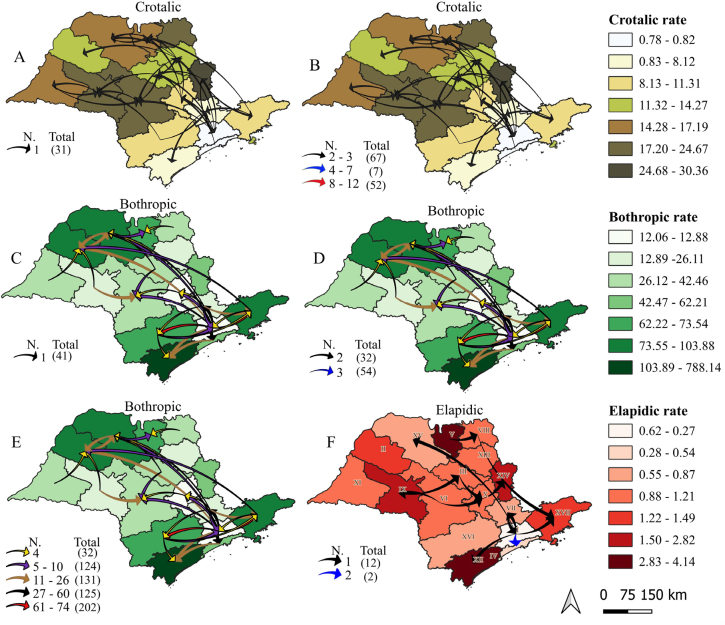
Distribution of non-autochthonous accidents in the state of São Paulo (2010–2022), according to the place of residence and the place of occurrence (represented by arrows), and incidence rate (per 100,000 inhabitants-year) of snakebite accidents, according to snake genus and Regional Health Departments (RHD).

Source: Notification Disease Information System (*Sistema de Informação de Agravos de Notificação* – SINAN).

Among the reported accidents, 2,210 (13.1%) of the *Bothrops* incidents, 521 (15.9%) of the *Crotalus* incidents, and 44 (13.2%) of the *Elapid* incidents were classified as non-autochthonous. In *Bothrops* accidents, the mean distance between the place of residence and the location of occurrence was 56.3 km, with a median of 29.4 km (Piracaia/Bragança Paulista), a minimum of 3.4 km (Franco da Rocha/Francisco Morato), and a maximum of 787.3 km (Teodoro Sampaio/Roseira). In *Crotalus* accidents, the mean distance was 43.2 km, with a median of 27.4 km (Taubaté/Terra Roxa), a minimum of 5.4 km (Franco da Rocha/Francisco Morato), and a maximum of 361.7 km (Lorena/Canas). In *Elapid* accidents, the average distance was 55.9 km, with a median of 29.1 km (Ituverava/Ipuã), a minimum of 9.8 km (Campinas/Valinhos), and a maximum of 496.8 km (São Paulo/Vitória Brazil). Of the 54 *Bothrops* deaths recorded, seven (13.0%) were non-autochthonous. Among the 18 *Crotalus* deaths, only one (5.57%) was classified as non-autochthonous (Figure 2).

Figure 2 illustrates the incidence rates for autochthonous accidents and, for non-autochthonous accidents, arrows on the maps indicate the flow between the RHD of residence and occurrence. *Bothrops* accidents were concentrated in the coastal, southern, southeastern, and northern regions, with particular emphasis on the RHD of Registro, Baixada Santista, Taubaté, Sorocaba, Araçatuba, São José do Rio Preto, Barretos, and São João da Boa Vista. In contrast, *Crotalus* accidents were more frequent in the central, northeastern, and northwestern regions of the state, with notable concentrations in the RHD of São João da Boa Vista, Bauru, Marília, Franca, Barretos, São José do Rio Preto, and Presidente Prudente. *Elapid* accidents occurred primarily in the RHD of Registro, Taubaté, São João da Boa Vista, Presidente Prudente, Barretos, and Araçatuba. In this context, the RHD of São João da Boa Vista exhibited high incidence rates for all three types of accidents.

In non-autochthonous accidents, the arrows on the maps indicate the directions and distances, showing that most incidents occurred, on average, 50 km from the residence. However, extreme cases were observed, with some accident records occurring as far as nearly 800 km away from the victim’s place of residence.

Figure 2A highlights 31 non-native *Crotalus* accidents, with a concentration of residents from RHD I — Greater São Paulo — who experienced accidents in RHDs in the central-west region. Figure 2B presents 67 records with 2–3 accidents, seven records with 4–7 accidents, and 52 records with 8–12 accidents. There is a concentration of residents from Greater São Paulo who had accidents in the RHDs of Sorocaba, Campinas, and Taubaté, as well as residents from the RHDs of Campinas, Araraquara, Marília, and Sorocaba who had accidents in the RHD of Bauru.

Figures 2C–2E display non-autochthonous *Bothrops* accidents, highlighting the greatest distances between the place of residence and the location of occurrence. Figure 2C shows 41 records of accidents with no apparent concentration. Figure 2D displays 32 records with two accidents and 54 records with three accidents, revealing two concentration patterns: the first in the RHD of Taubaté, Baixada Santista, and Registro, and the second in the RHD of Araçatuba and São José do Rio Preto, involving patients from various regions of the state. Figure 2E illustrates 32 records with four accidents, 124 records with 5–10 accidents, 131 with 11–26 accidents, 125 with 27–60 accidents, and 202 with 61–74 accidents. This map demonstrates that the RHD of Greater São Paulo had the highest number of residents who experienced accidents in other locations within the state. Figure 2F shows non-autochthonous *Elapid* accidents, among which no distinct concentration pattern was identified.

**Figure 3:**
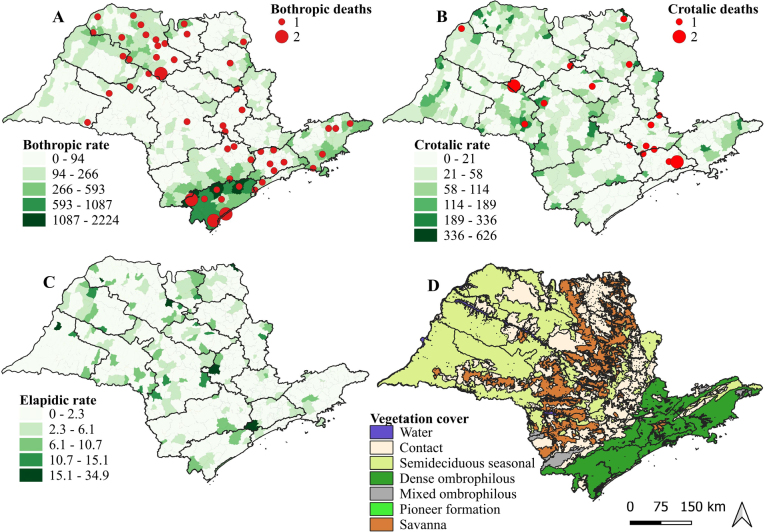
Distribution of autochthonous accidents and deaths in the state of São Paulo (2010–2022) according to local Bayesian incidence rates (per 100,000 inhabitants-year), by snake genus, municipality of occurrence, Regional Health Departments (RHD), and vegetation cover.

Source: Notification Disease Information System (*Sistema de Informação de Agravos de Notificação* – SINAN).

The Bayesian incidence rates of autochthonous snakebites were calculated by municipality, revealing distinct patterns. For *Bothrops* snakebites, the municipalities with the highest rates, in decreasing order, were: Iporanga, Sete Barras, Miracatu, Juquiá, and Barra do Turvo (Figure 3A). For *Crotalus* snakebites, the municipalities with the highest rates were Rifaina, São Francisco, São João das Duas Pontes, Cássia dos Coqueiros, and Santa Mercedes (Figure 3B). For *Elapid* snakebites, the municipalities with the highest rates were Juquitiba, São Lourenço da Serra, São Francisco, São Pedro, and Tabapuã (Figure 3C).

The distribution of *Bothrops* accidents by vegetation cover shows that 56.6% occurred in areas of dense ombrophilous forest, 20.4% in semi-deciduous seasonal forest, 16.9% in regions with mixed vegetation, 5.0% in savannas, and 1.1% in mixed ombrophilous forest. In *Crotalus* accidents, 36.0% occurred in areas of semi-deciduous seasonal forest, 28.0% in mixed vegetation, 21.3% in dense ombrophilous forest, 14.0% in savannas, and 0.7% in mixed ombrophilous forest. *Elapid* accidents presented a pattern similar to that of *Bothrops*, with 40.8% occurring in dense ombrophilous forest, 28.2% in semi-deciduous seasonal forest, 22.5% in mixed vegetation, 8.1% in savannas, and 0.3% in mixed ombrophilous forest (Figure 3D).

**Figure 4:**
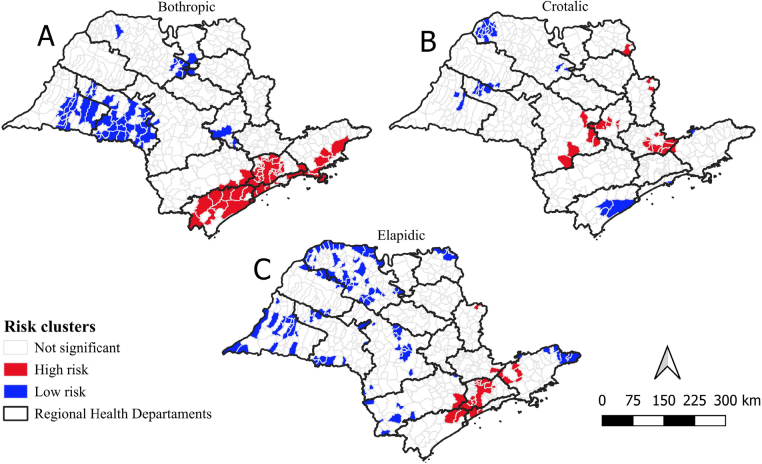
Distribution of high and low-risk clusters in the state of São Paulo (2010–2022) according to the Getis and Ord index (Gi*), the municipality of occurrence, and the type of snakebite accident.

Source: Notification Disease Information System (*Sistema de Informação de Agravos de Notificação* – SINAN).

Analysis of the Getis and Ord index (Gi*) shows that the highest risk of *Bothrops* accidents is concentrated in the southern, southeastern, and coastal areas of São Paulo (Figure 4A). For *Crotalus* accidents, the risk is highest in the central region, including Botucatu, Piracicaba, Limeira, Campinas, Rio Claro, Jaú, Franca, São José do Rio Pardo-Mococa, Amparo, Bragança Paulista, Jundiaí, and the northwest of São José dos Campos (Figure 4B). *Elapid* accidents present a high risk in areas that overlap with other types, primarily in Greater São Paulo, Registro, Santos, Sorocaba, São José dos Campos, and São José do Rio Pardo-Mococa (Figure 4C).

## DISCUSSION

Between 2010 and 2022, the majority of snakebites in São Paulo were caused by *Bothrops*, followed by *Crotalus* and *Elapids*. The highest prevalence was observed among men, adults, predominantly white individuals, and those with low levels of education. Most accidents occurred in urban areas, with treatment provided within one hour. However, deaths were more prevalent in rural areas, where treatment took longer. Although the mortality rate was low, deaths were more common among aged individuals. Geographically, in the state of São Paulo, *Bothrops* predominated in the south and southeast, *Crotalus* in the center and north, and *Elapids* throughout the state. Vegetation cover also influenced the distribution of accidents, varying according to the snake genus.

São Paulo is the 6^th^ state with the highest number of snakebite reports in Brazil^3^, with *Bothrops* being the most frequent species^7,20,21^. The high incidence of snakebite accidents may be related to the adaptation of snakes to different environments and the urbanization process, which brings these animals closer to urban areas. This phenomenon promotes the proliferation of rodents and other synanthropic animals, attracted by debris or shelters, thereby creating conditions conducive to the “urbanization of snakebite accidents”^7,22^.

Men were the most affected by snakebite accidents, a pattern that has been reported in the literature for decades and is often linked to work activities predominantly performed by men, such as agriculture, fishing, and livestock farming^23^. This observation may also explain the other social pattern identified in this study, where the highest incidence was recorded among individuals with lower levels of education^21,24^.

Unlike the national trend^3^, where black and mixed-race populations are more vulnerable to snakebite accidents, this study found a higher incidence among white individuals. This difference may be attributed to regional characteristics, such as a higher prevalence of white individuals engaging in risky activities or differences in access to and recording of health data. This finding underscores the importance of considering local specificities in the analysis of snakebite incidents.

The adult population (20–59 years) was the most affected, likely due to greater exposure in work activities. This increased exposure can lead to consequences such as temporary work loss during recovery or even permanent disability due to sequelae^25^.

Unlike what is generally observed in the literature, this study revealed that the largest number of snakebites occurred in urban areas. Two possible explanations for this phenomenon can be considered:

- inconsistency in notifications regarding the identification of the area of occurrence;
- adaptation of snakes to urban areas.

However, it is important to note that the significant difference between urban and rural populations makes direct comparison challenging. Ideally, exposure to the risk of snakebites should be assessed using the incidence rate in each population, but the information available in this study did not allow for such an analysis.

As described in the literature^26^, the highest frequency of deaths occurred in rural areas, where the time between the accident and treatment was longer. According to Gutiérrez et al.^27^, the time between poisoning and the first care is crucial and directly related to therapeutic success, as the serum neutralizes the toxins circulating in the blood. Therefore, the therapeutic path followed by patients significantly influences the speed at which treatment is initiated.

The number of accidents between 2010 and 2022 remained stable, consistent with previous studies^28^. However, there was a slight increase in accidents involving rattlesnakes, which may be linked to the replacement of semideciduous, mixed, and cerrado vegetation by pastures and agricultural areas. This environmental change expands the distribution of rattlesnakes to new ecological niches, where they can adapt quickly^29^.

Accidents were more frequent during hot and rainy periods, which aligns with findings from other studies^30,31^ that associate these seasons with favorable environmental conditions for snake reproduction, including gestation and the birth of young, due to the greater availability of resources^32^.

It has been observed that snakes of the *Bothrops* genus tend to inhabit regions with higher temperatures, high humidity, and high annual precipitation, characteristics typical of dense rainforests. However, the wide diversity of species in this genus allows them to adapt and survive in drier and/or colder regions, such as semideciduous seasonal forests, mixed forests, and savannah areas^33^. In contrast, snakes of the *Crotalus* genus prefer warmer and drier environments with sparse vegetation cover and smaller size^12,29^. The distribution of *Elapid* accidents, particularly those involving species of the *Micrurus* genus, reveals their presence throughout Brazil^34^. These accidents are often linked to handling, especially by children, contributing to a higher incidence among younger patients. The vibrant coloration and less intimidating behavior of these snakes tend to arouse curiosity, which increases the risk of handling and, consequently, accidents^35^.

A considerable number of non-native accidents were recorded, highlighting the importance of assessing severity based on the place of occurrence. This emphasizes the need for studies that investigate factors related to the location of the accident and the journey to the care unit, aiming to improve prevention measures and enhance assistance to victims.

The identification of high and low-risk clusters enabled the recognition of areas with greater or lesser potential for accidents based on the snake genus, revealing a spatial distribution closely aligned with the incidence rate. These findings corroborate previous studies^27,36^, particularly in regions identified in this research as having a high risk for Bothrops and Crotalus accidents.

Limitations include the quality of records and underreporting of accidents, which can reduce awareness of the true magnitude of the problem. Studies comparing the incidence of accidents by urban and rural areas and populations are recommended, as well as investigating factors associated with non-indigenous accidents and possible protections in low-risk areas. It is also essential to analyze the location of deaths related to these accidents.

By identifying the most affected population, seasonality, and locations with the highest risk of accidents, the results of this study can support managers in planning more effective actions for the prevention and management of snakebite accidents. This information allows efforts to be directed to more vulnerable populations, organize preventive actions before periods of highest incidence, and optimize resources through the training of professionals and adequate distribution of supplies, such as antivenoms, in regions of greatest risk. In addition, they favor the promotion of integrated actions, aligning health, environmental, and animal control management in a coordinated and comprehensive approach.

## Data Availability

The study was based on secondary data obtained from the “Prof. Alexandre Vranjac” Epidemiological Surveillance Center of the São Paulo State Health Secretariat. These data did not contain patient identification information, thus eliminating the need for approval by the ethics committee.
