# Supplementary material for: What are the characteristics and where is the highest risk of snakebite accidents in the state of São Paulo?
Source: Rev Bras Epidemiol. 2025 Jun 2;28:e250026. doi: 10.1590/1980-549720250026 (PMC12129242; doi:10.1590/1980-549720250026)
Supplement: Translation

# Translation: Quais características e onde há maior risco de acidente ofídico no estado de São Paulo?

**DOI:** 10.1590/1980-549720250026.2

**Authors:** Gisele Dias de Freitas, Alec Brian Lacerda, Thiago Salomão de Azevedo, Anderson de Oliveira, Roberta Maria Fernandes Spinola, Flávio Santos Dourado, Fan Hui Wen, Francisco Chiaravalloti-Neto

**Affiliations:** I Secretaria de Estado da Saúde de São Paulo, Coordination of Disease Control, “Prof. Alexandre Vranjac” Epidemiological Surveillance Center – São Paulo (SP), Brasil.; II Universidade de São Paulo, School of Public Health – São Paulo (SP), Brasil.; III Secretaria de Saúde do Município de Santa Bárbara d’Oeste – Santa Bárbara d’Oeste (SP), Brasil.; IV Ministério da Saúde, Health Surveillance and Environment Secretariat, General Coordination of Zoonoses and Vector-Borne Diseases Surveillance – Brasília (DF), Brasil.; V Instituto Butantan, Bioindustrial Center – São Paulo (SP), Brasil.

**Keywords:** Mordeduras de serpentes, Animais peçonhentos, Serpentes peçonhentas, Antivenenos, Imunização passiva

## Abstract

**Objetivos:**

Para compreender o padrão do ofidismo, o objetivo foi descrever os acidentes e óbitos por gênero de serpente, faixa etária, sexo, raça/cor, escolaridade, local de residência e ocorrência, sazonalidade e cobertura vegetal e identificar aglomerados espaciais com maior risco para acidentes no estado de São Paulo.

**Métodos:**

Estudo descritivo com dados do Sistema de Informação de Agravos de Notificação (SINAN), sobre acidentes ofídicos entre 2010 e 2022. Índice de Getis e Ord (Gi*) utilizados para a identificação de aglomerados de alto e baixo risco.

**Resultados:**

Acidentes botrópicos predominaram (61,5%), afetando homens (78,7%), brancos (64,7%), adultos entre 20 e 59 anos (67,1%) e pessoas com baixa escolaridade (63,4%). A maioria dos acidentes ocorreu em zonas urbanas (55,7%), enquanto óbitos foram mais frequentes em áreas rurais (68,1%), com maior atraso no atendimento. A incidência permaneceu estável, aumentando em períodos quentes e chuvosos. Muitos casos não foram autóctones, e o tipo de vegetação influenciou o padrão de acidentes: *Bothrops* em áreas densas e úmidas, *Crotalus* em regiões secas e abertas e *Micrurus* em ambas. A análise espacial destacou áreas de maior e menor risco, variando conforme o tipo de acidente.

**Conclusões:**

A identificação da população mais afetada, da sazonalidade e das áreas de maior risco fornece subsídios essenciais para ações preventivas e de manejo eficaz. Os achados podem orientar esforços para populações vulneráveis, antecipar estratégias preventivas em períodos de maior incidência e otimizar recursos, como a capacitação de profissionais e a distribuição de soros antiveneno nas regiões de maior risco.

## INTRODUÇÃO

Os acidentes ofídicos representam um problema relevante no Brasil e no mundo. Globalmente, estima-se a ocorrência de aproximadamente 5,4 milhões de acidentes e 137 mil mortes por ano^1^, com a possibilidade de que os números reais sejam ainda maiores^2^. Em 2022, o Brasil registrou 29.543 acidentes com serpentes e 94 óbitos^3^. Dentro da abordagem “Uma Só Saúde” (One Health), esse problema exige estratégia integrada, que leve em consideração as inter-relações entre saúde humana, animal e ambiental, visando a um enfrentamento eficaz e abrangente^4^.

No Brasil, esses incidentes são registrados no Sistema de Informação de Agravos de Notificação (SINAN) desde 1995, mas ganharam maior relevância em 2010, quando foram incluídos na Lista de Notificação Compulsória. Atualmente, os acidentes ofídicos estão entre os agravos mais frequentemente notificados no país^3^. Além disso, em 2017, foram reintegrados à lista de doenças tropicais negligenciadas pela Organização Mundial da Saúde (OMS)^5^. No país, as serpentes de importância médica pertencem às famílias Viperidae (*Crotalus* — cascavel, *Bothrops* — jararaca, *Lachesis* — surucucu) e Elapidae (*Micrurus* — corais), responsáveis pelos acidentes crotálicos, botrópicos, laquéticos e elapídicos, respectivamente^6^.

No estado de São Paulo, há grande diversidade de paisagens naturais e condições climáticas, como as encontradas nos biomas Mata Atlântica e Cerrado, que abrigam ampla variedade de serpentes. Entre elas, destacam-se as espécies peçonhentas dos gêneros *Bothrops*, *Crotalus* e *Micrurus*, que são as principais causadoras de acidentes ofídicos na região. Embora, tradicionalmente, os acidentes ocorram com mais frequência em áreas rurais, a incidência nas zonas urbanas tem aumentado. Esse aumento é impulsionado pela expansão desordenada das cidades e pelo acúmulo de lixo, que atrai roedores — principais presas das serpentes. Esse cenário reforça a necessidade de manejo ambiental eficaz e de maior conscientização pública, a fim de reduzir os riscos de contato com esses animais^7^.

Diante do exposto e da necessidade de compreender o padrão do ofidismo em São Paulo, o objetivo deste estudo foi analisar os acidentes causados por serpentes peçonhentas e os óbitos relacionados, por meio da avaliação das características da população afetada (faixa etária, sexo, raça/cor e escolaridade), espécies envolvidas, sazonalidade (seca ou chuva) e localização dos acidentes considerando-se a cobertura vegetal. A identificação de locais de maior risco pode contribuir para orientar ações de saúde pública, como estratégias preventivas, alocação de recursos e planejamento de atendimento em áreas prioritárias.

## MÉTODOS

Foi realizado estudo descritivo sobre acidentes ofídicos, no estado de São Paulo, entre 2010 e 2022, com dados extraídos do Sistema de Informação de Agravos de Notificação (SINAN)^8^. As informações foram selecionadas das notificações dos acidentes por animais peçonhentos, que é composto dos acidentes causados por abelhas, aranhas, escorpiões, lagartas, serpentes e outros animais. Dentre os acidentes ofídicos, foram selecionados aqueles causados por serpentes peçonhentas (acidentes botrópicos, crotálicos, elapídicos e laquéticos). Os laquéticos não foram considerados neste trabalho, pois não há esse gênero de serpente, em vida livre, no estado de São Paulo^9^.

Para avaliar as características sociodemográficas dos acidentados e da localização dos acidentes, utilizaram-se as variáveis: idade, sexo, raça/cor, escolaridade, data do acidente, município de residência e ocorrência, zona de ocorrência, tempo entre acidente e atendimento e evolução do caso.

Taxas de incidência, mortalidade e letalidade foram calculadas por gênero de serpente, faixa etária (0–9, 10–19, 20–59 e 60+) e sexo. A taxa de incidência anual foi obtida dividindo-se o número de acidentes pela população de 2016, dividindo o resultado por 13 (anos do estudo) e multiplicando por 1 milhão. A taxa de mortalidade seguiu o mesmo cálculo, substituindo acidentes por óbitos. A letalidade foi calculada dividindo-se óbitos por acidentes e multiplicando por 100. Na série histórica, utilizou-se escala logarítmica por conta da amplitude entre tipos de acidentes, e na sazonalidade a representação foi em porcentagens pelo mesmo motivo.

As taxas de incidência da distribuição espacial foram calculadas utilizando taxas bayesiana empíricas locais^10^, tendo como base apenas os acidentes autóctones segundo tipo de acidente e município de ocorrência. Consideraram-se acidentes autóctones, aqueles cujo município ou Departamentos Regionais de Saúde (DRS) de residência e de ocorrência eram iguais. Para os não autóctones, foi avaliada a distância entre o município de residência e de ocorrência de cada acidente, calculada por meio da fórmula de cálculo de soluções diretas e inversas de geodésicas no elipsoide^11^.

As informações demográficas e de vegetação foram adquiridas no Instituto Brasileiro de Geografia e Estatística (IBGE). As coberturas vegetais avaliadas foram: florestas ombrófilas densas e mistas, florestas estacionais semidecidual, vegetação mista e savana^12^.

O índice de Getis e Ord (Gi*) foi utilizado para analisar localmente a associação espacial baseada num indicador de concentração de riscos para os acidentes ofídicos, aplicando-se o ajuste da “razão de falsas descoberta/ taxas de falsas descobertas” (*false discovery rate* — FDR)^13^. Foram considerados acidentes segundo gênero de serpente e município de ocorrência. O cálculo do peso da matriz de vizinhança foi feito pela técnica de pesos espaciais de contiguidade difusa (*fuzzy contiguity spatial weights*), utilizando os pesos espaciais de contiguidade difusa do pacote Pysal para incluir espacialmente o município de Ilhabella ao Estado de São Paulo^13^. Os resultados foram avaliados com a identificação de aglomerados de alto e baixo risco para acidente ofídico.

Os *softwares* utilizados neste estudo foram: GeoDa^14^, Python^15^, Qgis^16^, R-4.3.2.^17^, SatScan^18^ e Tabwin^19^.

## RESULTADOS

No estado de São Paulo, entre 2010 e 2022, dos 27.777 acidentes com serpentes registrados, 17.740 (63,9%) foram causados por espécies peçonhentas, sendo 61,5% botrópicos, 12,1% crotálicos e 1,2% elapídico (Tabela 1). A incidência de acidentes botrópicos foi 28,8 casos por 1 milhão de habitantes-ano, com homens 2,9 vezes mais afetados. Em adultos acima de 20 anos, a taxa foi 1,6 vez maior que em crianças. Os acidentes crotálicos apresentaram 5,6 casos por 1 milhão de habitantes-ano, com predominância três vezes maior em homens e 2,7 vezes em adultos. Os acidentes elapídicos registraram 0,6 caso por 1 milhão de habitantes-ano, também com predomínio 2,9 vezes em homens e adultos (Tabela 1).

**Tabela 1:** Distribuição dos acidentes ofídicos no Estado de São Paulo (2010–2022), por tipo de acidente, sexo, faixa etária, taxa de incidência e mortalidade (por 1.000.000 habitantes/ano) e porcentagem de letalidade anual.

Tipo de Acidente	Sexo	Variável	Faixa Etária (Anos)				Total
			0 A 9	10 A 19	20 A 59	60 Ou +	
Botrópico	Homens	Incidência	16,51	39,39	53,11	53,61	46,57
		Casos	623	1.456	9.026	2.197	13.302
		Mortalidade	0,03	0,00	0,11	0,59	0,15
		Óbitos	1	0	19	24	44
		Letalidade	0,01	0,00	0,02	0,08	0,03
	Mulheres	Incidência	8,36	13,35	12,62	11,63	12,02
		Casos	301	475	2.216	629	3621
		Mortalidade	0,00	0,00	0,02	0,13	0,04
		Óbitos	0	0	4	7	11
		Letalidade	0,00	0,00	0,01	0,09	0,02
	Total	Incidência	12,53	26,61	32,53	29,73	28,83
		Casos	924	1.931	11.242	2.826	16.923
		Mortalidade	0,01	0,00	0,07	0,33	0,09
		Óbitos	1	0	23	31	55
		Letalidade	0,01	0,00	0,02	0,08	0,03
Crotálico	Homens	Incidência	1,96	6,65	10,89	11,22	9,21
		Casos	74	246	1.850	460	2.630
		Mortalidade	0,03	0,03	0,05	0,17	0,06
		Óbitos	1	1	8	7	17
		Letalidade	0,10	0,03	0,03	0,12	0,05
	Mulheres	Incidência	1,42	1,74	2,50	1,79	2,15
		Casos	51	62	439	97	649
		Mortalidade	0,00	0,00	0,00	0,02	0,00
		Óbitos	0	0	0	1	1
		Letalidade	0,000	0,000	0,000	0,079	0,012
	Total	Incidência	1,70	4,24	6,62	5,86	5,59
		Casos	125	308	2.289	557	3.279
		Mortalidade	0,01	0,01	0,02	0,08	0,03
		Óbitos	1	1	8	8	18
		Letalidade	0,062	0,025	0,027	0,110	0,042
Elapídico	Homens	Incidência	0,53	0,84	1,00	0,41	0,83
		Casos	20	31	170	17	238
		Óbitos	0	0	0	0	0
	Mulheres	Incidência	0,25	0,48	0,38	0,04	0,32
		Casos	9	17	67	2	95
		Óbitos	0	0	0	0	0
	Total	Incidência	0,39	0,66	0,69	0,20	0,57
		Casos	29	48	237	19	333
		Óbitos	0	0	0	0	0
Total	Homens	Incidência	19,00	46,88	65,00	65,25	56,61
		Casos	717	1.733	11.046	2.674	16.170
		Mortalidade	0,05	0,03	0,16	0,76	0,21
		Óbitos	2	1	27	31	61
		Letalidade	0,021	0,004	0,019	0,089	0,029
	Mulheres	Incidência	10,03	15,57	15,50	13,46	14,49
		Casos	361	554	2.722	728	4.365
		Mortalidade	0,00	0,00	0,02	0,15	0,04
		Óbitos	0	0	4	8	12
		Letalidade	0,000	0,000	0,011	0,085	0,021
	Total	Incidência	14,62	31,52	39,84	35,78	34,99
		Casos	1.078	2.287	13.768	3.402	20.535
		Mortalidade	0,03	0,01	0,09	0,41	0,12
		Óbitos	2	1	31	39	73
		Letalidade	0,014	0,003	0,017	0,088	0,027

Fonte: Sistema de Informação de Agravos de Notificação (SINAN).

Diante da semelhança observada nos padrões dos resultados referentes à raça/cor e escolaridade, avaliados separadamente para cada gênero de serpente, optou-se por apresentar essas análises de forma agregada entre os gêneros. A incidência anual por raça/cor foi: 20,98 casos por 1 milhão de habitantes-ano em brancos, 8,7 em pardos, 2,1 em pretos e 0,3 em amarelos e indígenas. Quanto à escolaridade, predominaram pacientes com ensino fundamental II (18,6%), seguido por ensino médio (17,8%) e ensino fundamental I (16,5%). Apenas 3,5% ocorreram em pessoas com ensino superior e 1,7% entre analfabetos. Em 40,4% das notificações, a escolaridade não foi informada. Dos acidentes, 55,7% ocorreram em zonas urbanas, 37,6% rurais e 2,1% periurbanas, com 4,6% sem localização informada. Quanto ao tempo até o tratamento: 49,2% foram atendidos em até uma hora, 27,2% em 1–3 horas, 9,7% em 3–6 horas, 2,9% em 6–12 horas, 2,1% em 12–24 horas e 2,8% após 24 horas. Em 6,1% dos casos, o tempo não foi registrado.

Entre 17.740 acidentes e 73 óbitos (55 botrópicos e 18 crotálicos), a taxa de mortalidade foi 0,1 óbito por 1 milhão de habitantes-ano, com letalidade geral de 0,03%. Nos acidentes botrópicos, a mortalidade foi 3,7 vezes maior em idosos comparados a adultos e 20 vezes maior que em crianças. Nos homens, a mortalidade foi três vezes maior, sendo 4,4 vezes maior em idosos *versus* adultos e 8,9 vezes mais frequente que em crianças. Nos acidentes crotálicos, a taxa foi de 0,03 óbito por 1 milhão de habitantes-ano, 2,7 vezes maior em idosos e 4,5 vezes maior que em crianças/adolescentes. A mortalidade em homens foi 19 vezes superior à de mulheres (Tabela 1). Em razão da similaridade entre os óbitos, as análises foram agrupadas. Quanto à raça/cor: 72,2% eram brancos, 15,3% pardos, 6,9% pretos e 5,6% sem informação. Com relação à escolaridade: 20,8% tinham ensino fundamental II, 18,1% fundamental I, 4,2% eram analfabetos, 4,2% possuíam ensino médio e superior e 48,6% não tinham informação. Dos óbitos, 68,1% ocorreram em áreas rurais, 18,1% urbanas, 6,9% periurbanas e 6,9% sem informação. O tempo até o atendimento foi: 31,9% em até uma hora, 23,6% em 3–6 horas, 15,3% em 1–3 horas, 12,5% após 24 horas, 6,9% em 12–24 horas, 4,2% em 6–12 horas e 5,6% não tinham registro (Tabela 1).

As notificações de acidentes botrópicos e crotálicos mantiveram-se estáveis ao longo do tempo, enquanto os acidentes elapídicos apresentaram redução em 2015 (Figura 1A). Durante o inverno paulista (junho a agosto), houve menos notificações de acidentes botrópicos e elapídicos, enquanto os crotálicos permaneceram mais constantes (Figura 1B). Em acidentes botrópicos, 2016 registrou o maior número de óbitos (7), seguido por 2019–2021 (6/ano) e 2011 (5). Março teve mais óbitos (15), seguido por setembro-novembro (6/mês). Nos acidentes crotálicos, 2011, 2012 e 2017 tiveram mais registros (3/ano), com fevereiro apresentando maior frequência (quatro óbitos).

**Figura 1:** Distribuição do número* de acidentes ofídicos no estado de São Paulo (2010–2022), segundo o gênero de serpente, ano (temporalidade) e mês (sazonalidade) de ocorrência.

*Na série histórica (A), utilizou-se escala logarítmica para apresentar o número de acidentes, em razão da grande diferença de amplitude entre os três tipos de acidentes. Na sazonalidade (B), a representação foi em porcentagens pelo mesmo motivo.

Fonte: Sistema de Informação de Agravos de Notificação (SINAN).

Entre os acidentes notificados, 2.210 (13,1%) dos botrópicos, 521 (15,9%) dos crotálicos e 44 (13,2%) dos elapídicos não foram autóctones. Nos botrópicos, a distância média entre o local de residência e de ocorrência foi de 56,3 km, com mediana de 29,4 km (Piracaia/Bragança Paulista), mínima de 3,4 km (Franco da Rocha/Francisco Morato) e máxima de 787,3 km (Teodoro Sampaio/Roseira). Nos crotálicos, a média foi 43,2 km, mediana 27,4 km (Taubaté/Terra Roxa), mínima 5,4 km (Franco da Rocha/Francisco Morato) e máxima 361,7 km (Lorena/Canas). Já nos elapídicos, a distância média foi 55,9 km, mediana 29,1 km (Ituverava/Ipuã), mínima 9,8 km (Campinas/Valinhos) e máxima 496,8 km (São Paulo/Vitória Brasil). Dos 54 óbitos botrópicos registrados, sete (13,0%) não foram autóctones. Já entre os 18 óbitos crotálicos, apenas um (5,57%) foi classificado como não autóctone (Figura 2).

**Figura 2:** Distribuição dos acidentes não autóctones no estado de São Paulo (2010–2022), segundo o local de residência e o local de ocorrência (representado pelas setas), e taxa de incidência (por 100.000 habitantes-ano) dos acidentes ofídicos, de acordo com o gênero de serpente e os Departamentos Regionais de Saúde (DRS).

Fonte: Sistema de Informação de Agravos de Notificação (SINAN).

A Figura 2 apresenta, para os acidentes autóctones, as taxas de incidência e, para os não autóctones, setas nos mapas indicando o fluxo entre os DRS de residência e ocorrência. Os botrópicos concentraram-se nas regiões litorânea, sul, sudeste e norte, com destaque para os DRS de Registro, Baixada Santista, Taubaté, Sorocaba, Araçatuba, São José do Rio Preto, Barretos e São João da Boa Vista. Por outro lado, os acidentes crotálicos foram mais frequentes nas regiões central, nordeste e noroeste do estado, destacando-se os DRS de São João da Boa Vista, Bauru, Marília, Franca, Barretos, São José do Rio Preto e Presidente Prudente. Já os elapídicos ocorreram principalmente nos DRS de Registro, Taubaté, São João da Boa Vista, Presidente Prudente, Barretos e Araçatuba. Nesse contexto, o DRS de São João da Boa Vista apresentou altas taxas de incidência para os três tipos de acidentes.

Nos acidentes não autóctones, as setas nos mapas indicam direções e distâncias, mostrando que a maioria ocorreu, em média, a 50 km da residência. Entretanto, houve casos extremos, com registros de acidentes a quase 800 km de distância do local de residência do acidentado.

A Figura 2A destaca os 31 acidentes crotálicos não autóctones, com concentração de moradores do DRS I — Grande São Paulo — que se acidentaram em DRS da região centro-oeste. Já a Figura 2B apresenta 67 registros com 2–3 acidentes, sete registros com 4–7 e 52 registros com 8–12 acidentes. Observa-se uma concentração de residentes da Grande São Paulo que se acidentaram nos DRS de Sorocaba, Campinas e Taubaté, além de moradores dos DRS de Campinas, Araraquara, Marília e Sorocaba que se acidentaram no DRS de Bauru.

As Figuras 2C–2E apresentam os acidentes botrópicos não autóctones, destacando as maiores distâncias entre o local de residência e de ocorrência. Na Figura 2C estão representados 41 registros de acidentes sem concentração aparente. Na Figura 2D aparecem 32 registros de dois acidentes e 54 registros de três acidentes, com dois padrões de concentração: o primeiro nos DRS de Taubaté, Baixada Santista e Registro, e o segundo nos DRS de Araçatuba e São José do Rio Preto, envolvendo pacientes provenientes de diversas regiões do estado. Já na Figura 2E são apresentados 32 registros de quatro acidentes, 124 registros de 5–10 acidentes, 131 de 11–26 acidentes, 125 de 27–60 acidentes e 202 de 61–74 acidentes. Este último mapa mostra que a DRS da Grande São Paulo teve o maior número de residentes que se acidentaram em outras localidades do estado. A Figura 2F apresenta os acidentes elapídicos não autóctones, entre os quais não foi identificado nenhum padrão de concentração.

As taxas bayesianas de incidência dos acidentes ofídicos autóctones foram calculadas por município, revelando padrões distintos. Para os acidentes botrópicos, os municípios com as maiores taxas, em ordem decrescente, foram: Iporanga, Sete Barras, Miracatu, Juquiá e Barra do Turvo (Figura 3A). Nos acidentes crotálicos, destacaram-se Rifaina, São Francisco, São João das Duas Pontes, Cássia dos Coqueiros e Santa Mercedes (Figura 3B). Já para os elapídicos, os municípios com maiores taxas foram Juquitiba, São Lourenço da Serra, São Francisco, São Pedro e Tabapuã (Figura 3C).

**Figura 3:** Distribuição dos acidentes autóctones e óbitos no estado de São Paulo (2010–2022) segundo as taxas bayesianas locais de incidência (por 100.000 habitantes-ano), de acordo com o gênero de serpente, o município de ocorrência, os Departamentos Regionais de Saúde (DRS) e a cobertura vegetal.

Fonte: Sistema de Informação de Agravos de Notificação (SINAN).

A distribuição dos acidentes botrópicos por cobertura vegetal mostra que 56,6% ocorreram em áreas de floresta ombrófila densa, 20,4% em floresta estacional semidecidual, 16,9% em regiões com vegetação mista, 5,0% em savanas e 1,1% em floresta ombrófila mista. Nos acidentes crotálicos, 36,0% aconteceram em áreas de floresta estacional semidecidual, 28,0% em vegetação mista, 21,3% em floresta ombrófila densa, 14,0% em savanas e 0,7% em floresta ombrófila mista. Já os acidentes elapídicos apresentaram um padrão semelhante ao dos botrópicos, com 40,8% ocorrendo em floresta ombrófila densa, 28,2% em floresta estacional semidecidual, 22,5% em vegetação mista, 8,1% em savanas e 0,3% em floresta ombrófila mista (Figura 3D).

A análise do índice de Getis e Ord (Gi*) mostra que o maior risco de acidentes botrópicos concentra-se no sul, sudeste e litoral de São Paulo (Figura 4A). Para acidentes crotálicos, o risco é maior na região central, incluindo Botucatu, Piracicaba, Limeira, Campinas, Rio Claro, Jaú, Franca, São José do Rio Pardo-Mococa, Amparo, Bragança Paulista, Jundiaí e noroeste de São José dos Campos (Figura 4B). Os acidentes elapídicos apresentam alto risco em áreas que se sobrepõem aos outros tipos, principalmente na Grande São Paulo, Registro, Santos, Sorocaba, São José dos Campos e São José do Rio Pardo-Mococa (Figura 4C).

**Figura 4:** Distribuição dos aglomerados de alto e baixo risco no estado de São Paulo (2010–2022) segundo o índice de Getis e Ord (Gi*), o município de ocorrência e o tipo de acidente ofídico.

Fonte: Sistema de Informação de Agravos de Notificação (SINAN).

## DISCUSSÃO

Entre 2010 e 2022, a maioria dos acidentes ofídicos em São Paulo foi botrópico, seguido por crotálico e elapídico. A maior prevalência ocorreu entre homens, adultos, principalmente brancos e com baixa escolaridade. A maioria dos acidentes aconteceu em áreas urbanas, com atendimento em até uma hora. No entanto, nos óbitos os acidentes foram mais prevalentes em zona rural, onde o tratamento demorou mais. Embora a mortalidade tenha sido baixa, os óbitos foram mais comuns entre idosos. Geograficamente, no estado de São Paulo, os botrópicos predominavam no sul e sudeste, os crotálicos no centro e norte e os elapídicos por todo o estado. A cobertura vegetal também influenciou a distribuição dos acidentes, variando conforme o gênero de serpente.

São Paulo é o 6º estado que mais notifica acidentes ofídicos no Brasil^3^, sendo os acidentes botrópicos os mais frequentes^7,20,21^. A alta incidência dos acidentes pode estar relacionada à adaptação das serpentes a diferentes ambientes e ao processo de urbanização, que aproxima esses animais das áreas urbanas. Esse fenômeno favorece a proliferação de roedores e outros animais sinantrópicos, atraídos por detritos ou abrigos, criando condições propícias para a “urbanização dos acidentes ofídicos”^7,22^.

Os homens foram os mais acometidos por acidentes ofídicos, um padrão já reportado na literatura há décadas e frequentemente associado às atividades laborais predominantemente desempenhadas por eles, como agricultura, pesca e agropecuária^23^. Essa observação também pode explicar o outro padrão social observado neste estudo, no qual a maior incidência foi registrada entre indivíduos com menor nível de escolaridade^21,24^.

Diferentemente do padrão nacional^3^, em que populações negras e pardas são mais vulneráveis a acidentes ofídicos, este estudo apontou maior acometimento entre pacientes brancos. Essa diferença pode estar associada às características regionais, como maior presença de pessoas brancas em atividades de risco ou diferenças no acesso e registro de dados de saúde. Esse achado reforça a importância de considerar especificidades locais.

A população adulta (20–59 anos) foi a mais afetada, o que pode ser atribuído à maior exposição desse grupo nas atividades laborais, resultando em consequências como a perda temporária de trabalho durante a recuperação ou até mesmo incapacidade permanente devida a sequelas^25^.

Diferentemente do que é geralmente observado na literatura, o presente estudo revelou que o maior número de acidentes ofídicos ocorreu em zonas urbanas. Duas possíveis explicações para esse fenômeno podem ser consideradas:

- inconsistência nas notificações quanto à identificação da zona de ocorrência;
- adaptação das serpentes às áreas urbanizadas.

Contudo, é importante observar que a diferença significativa entre as populações urbana e rural dificulta uma comparação direta. O ideal seria avaliar a exposição ao risco de acidentes ofídicos por meio da taxa de incidência em cada população, mas as informações disponíveis neste estudo não permitiram essa análise.

Já entre os óbitos, assim como é descrito na literatura^26^, a maior frequência dos acidentes aconteceu na zona rural, e o tempo entre o atendimento e o tratamento foi mais prolongado. Segundo Gutiérrez et al.^27^, o tempo entre o envenenamento e o primeiro atendimento é crucial e está diretamente relacionado ao sucesso terapêutico, uma vez que o soro neutraliza as toxinas circulantes no sangue. Dessa forma, o percurso terapêutico seguido pelos pacientes tem grande influência na rapidez com que o tratamento é iniciado.

O número de acidentes entre 2010 e 2022 manteve-se estável e está de acordo com estudos anteriores^28^. No entanto, observou-se discreto aumento nos acidentes envolvendo cascavéis, o que pode estar relacionado à substituição da vegetação semidecidual, mista e de cerrado por pastagens e áreas agrícolas. Essa mudança expande a distribuição das cascavéis para novos nichos ecológicos, onde elas se adaptam rapidamente^29^.

Os acidentes foram mais frequentes nos períodos quentes e chuvosos, o que está em concordância com outros estudos^30,31^ que associam esses períodos às condições ambientais favoráveis para a reprodução das serpentes, incluindo gestação e nascimento dos filhotes, dada a maior disponibilidade de recursos^32^.

Observou-se que as serpentes do gênero *Bothrops* tendem a ocupar regiões com temperaturas mais elevadas, alta umidade e elevada precipitação anual, características típicas de florestas ombrófilas densas. Contudo, a ampla diversidade de espécies nesse gênero permite que elas também se adaptem e sobrevivam em regiões mais secas e/ou frias, como florestas estacionais semideciduais, florestas mistas e áreas de savana^33^. Por outro lado, as serpentes do gênero *Crotalus* preferem ambientes mais quentes e secos, com cobertura vegetal esparsa e de menor porte^12,29^. A distribuição dos acidentes elapídicos, conforme descrito na literatura, revela a presença de espécies do gênero *Micrurus* em todo o território brasileiro^34^. Esses acidentes estão frequentemente associados ao manuseio, especialmente por crianças, o que contribui para a maior incidência entre os pacientes mais jovens. A coloração vibrante e o comportamento menos intimidador dessas serpentes tendem a despertar curiosidade, aumentando o risco de manipulação e, consequentemente, de acidentes^35^.

Registrou-se um número considerável de acidentes não autóctones, destacando-se a importância de avaliar o agravo com base no local de ocorrência. Isso reforça a necessidade de estudos que investiguem os fatores relacionados ao local onde o acidente aconteceu e ao deslocamento até a unidade de atendimento, visando melhorar as medidas de prevenção e a assistência às vítimas.

Os aglomerados de alto e baixo risco permitiram identificar áreas com maior ou menor potencial para acidentes, segundo o gênero de serpente, mostrando uma distribuição espacial semelhante à taxa de incidência. Esses resultados estão alinhados com a literatura^27,36^, especialmente nas regiões apontadas neste estudo como de alto risco para acidentes botrópicos e crotálicos.

Entre as limitações, destacam-se a qualidade dos registros e a subnotificação de acidentes, que podem reduzir a percepção da real magnitude do problema. Recomendam-se estudos comparando a incidência de acidentes por zona e populações urbana e rural, além de investigar fatores associados a acidentes não autóctones e possíveis proteções em áreas de baixo risco. Também é essencial analisar o local dos óbitos relacionados a esses acidentes.

Ao identificar a população mais afetada, a sazonalidade e os locais com maior risco de acidentes, os resultados deste estudo podem subsidiar os gestores no planejamento de ações mais eficazes para a prevenção e o manejo dos acidentes ofídicos. Essas informações permitem direcionar esforços para populações mais vulneráreis, organizar ações preventivas antes dos períodos de maior incidência e otimizar recursos por meio da capacitação de profissionais e distribuição adequada de insumos, como soros antivenenos, em regiões de maior risco. Além disso, favorecem a promoção de ações integradas, alinhando as gestões de saúde, meio ambiente e controle animal, em uma abordagem coordenada e abrangente.

## Data Availability

o estudo baseou-se em dados secundários obtidos do Centro de Vigilância Epidemiológica “Prof. Alexandre Vranjac” da Secretaria Estadual de Saúde de São Paulo. Esses dados não continham informações de identificação dos pacientes, portanto, dispensaram a necessidade de aprovação pelo comitê de ética.
